# 

**Published:** 1868-03

**Authors:** 


### Books and Pamphlets Received.

The Diagnosis, Pathology and Treatment of Diseases of Women, including the Diagnosis of Pregnancy. By Graily Hewitt, M. D., London, F. R. C. P., etc. First American from the second London edition, revised and enlarged, with one hundred and sixteen illustrations. Philadelphia: Lindsay & Blakiston, 1868. For sale by Theodore Butler.

A Practical Treatise on the Diseases of Women. By T. Gaillard Thomas, M. D., Professor of Obstetrics and Diseases of Women and Children in the College of Physicians and Surgeons, New York, etc., with two hundred and nineteen illustrations. Philadelphia: Henry C. Lea, 1868. For sale by Breed, Lent & Co.

The Principles and Practice of Obstetrics. By Gunning S. Bedford, A. M., M. D., Professor of Obstetrics, the Diseases of Women and Children, and Clinical Obstetrics in the University of New York, etc., illustrated by four colored lithographic plates and ninety-one wood engravings. Fourth edition, carefully revised throughout and enlarged. New York: Wm. Wood & Co., 1868. From the author.

Atlas of Venereal Diseases. By A. Cullerier, Surgeon to the Hôspital du Midi, etc. Translated from the French, with notes and additions, by Freeman J. Bumstead, M. D., Professor of Venereal Diseases in the College of Physicians and Surgeons, New York, etc., with about one hundred and fifty beautifully colored figures on twenty-six plates. Part 1, to be completed in five parts. Philadelphia: Henry C. Lea, 1868. For sale by Theodore Butler.

Address before the Philadelphia County Medical Society, delivered January 23, 1867, agreeable to a provision of the Constitution. By Wm. Mayburry, M. D., at the close of his official term as President. Published by order of the Society.

